# Implications of intestinal microecology and immune function alterations for immunotherapy outcomes in advanced unresectable lung adenocarcinoma

**DOI:** 10.1111/crj.13762

**Published:** 2024-04-29

**Authors:** Shuang He, Jin Tian, Jianhua Zang, Lin Long, Peng Liu, Yexi Zhang, Jun Xiao

**Affiliations:** ^1^ The First Clinical College of Shandong University of Traditional Chinese Medicine Jinan China; ^2^ Oncology Center I Department Qingdao Hiser Hospital Affiliated of Qingdao University (Qingdao Traditional Chinese Medicine Hospital) Qingdao China; ^3^ Department of Radiotherapy for Oncology Qingdao Hiser Hospital Affiliated of Qingdao University (Qingdao Traditional Chinese Medicine Hospital) Qingdao China; ^4^ Rehabilitation Centre of Acupuncture and Massage Qingdao Hiser Hospital Affiliated of Qingdao University (Qingdao Traditional Chinese Medicine Hospital) Qingdao China

**Keywords:** 16S rRNA gene sequencing, immunotherapy, intestinal microecology, lung adenocarcinoma

## Abstract

**Objective:**

This investigation aims to explore alterations in intestinal microecology and immune function among patients with advanced, unresectable lung adenocarcinoma undergoing different outcomes from immunotherapy.

**Methods:**

A cohort of 30 patients diagnosed with advanced unresectable lung adenocarcinoma received sintilimab immunotherapy as a monotherapy. Post four treatment cycles, efficacy was assessed, leading to the segregation of patients into two distinct cohorts: those responsive to treatment and those nonresponsive. Analysis involved observing variations in the abundance, distribution, and composition of fecal intestinal microorganisms pretreatment and posttreatment via 16S rRNA gene sequencing.

**Results:**

In this study involving 30 advanced lung adenocarcinoma patients, significant observations were made regarding the impact of immunotherapy on immune function and the gut microbiome composition. Patients were divided into treatment and control groups, revealing that immunotherapy led to a significant increase in CD4+ T cells and a decrease in CD8+ T cells among the treatment‐responsive individuals, indicating an enhanced immune response. Furthermore, an in‐depth analysis of the gut microbiome showed an increase in diversity and abundance of beneficial bacteria such as Faecalibacterium and Subdoligranulum in the treatment group. These findings highlight the dual effect of immunotherapy on modulating immune function and altering gut microbiome diversity, suggesting its potential therapeutic benefits in improving the health status of patients with advanced lung adenocarcinoma.

**Conclusion:**

The structuring of gut flora plays a pivotal role in augmenting the efficacy of anti‐tumor immunotherapy, underscoring the interplay between intestinal microecology and immune response in cancer treatment outcomes.

## Graphical abstract

Venn diagram illustrating the distribution of OTUs. Each color represents a different sample. The investigation produced a total of 2705 operational taxonomic units (OTUs), with 2595 remaining after normalization. Within these, 146 OTUs were identified in MYA1, 58 in MYA2, 86 in MYB1, and an elevated number of 462 in MYB2, totaling 748 OTUs across all groups.

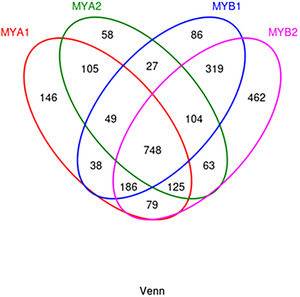

## 1 INTRODUCTION

Lung cancer remains a dominant malignant tumor globally, with its incidence rates escalating annually.^1^ In China, the prevalence and mortality rates of lung cancer rank among the highest worldwide. Current statistics reveal that nearly 2 million new lung cancer cases emerge globally each year.^2^ Regrettably, most patients receive a diagnosis at advanced stages, leading to a dismal 5‐year survival rate of merely 18%.^3^ Consequently, lung cancer research persists as a pivotal scientific endeavor for medical researchers.

The gut microbiota plays multifaceted roles in nutrient absorption, body metabolism, and the regulation of systemic inflammation and immune functions.^4^, ^5^, ^6^ These mechanisms impact the body's internal milieu, thereby influencing tumor progression. Recent insights suggest^7^, ^8^ that maintaining intestinal homeostasis could significantly enhance cancer treatment efficacy and affect tumor dynamics. The advent of microbiomics and advanced sequencing technologies has popularized microbial amplicon sequencing. Notably, 16S rRNA sequencing has become a key research tool, focusing on the analysis of microbial community distribution, abundance changes, and composition by amplifying a specific DNA region of environmental microorganisms. Hence, this method is pivotal in elucidating the correlation between microbial diversity and diseases.

The medical concept of the “lung‐gut axis” delineates the relationship between lung and intestinal diseases. The gut and lung microbiotas are intimately connected via lymphatic and blood circulations, with gut microbes capable of modulating the immune and defense responses of the lungs through this axis. Cremonesi et al.^9^ employed quantitative reverse transcription PCR to assess the expression of genes encoding immune cell markers, chemokines, and bacterial 16S ribosomal RNA (16S rRNA) in colorectal cancer (CRC) specimens and adjacent nontumor tissues. They observed that intestinal flora stimulates chemokine production in CRC cells, facilitating T‐cell infiltration into tumor tissues and bolstering the anti‐tumor response.

Immunotherapy, though an innovative anti‐tumor treatment, benefits only a limited patient subset. Variability in treatment response is ascribed to different tumor types and patient‐specific differences, closely associated with intestinal flora composition. Studies have indicated that targeted interventions on intestinal flora can amplify the defense and therapeutic outcomes in patients.^10^ Noteworthy is the engagement of intestinal flora in anti‐tumor mechanisms across chemotherapy, radiotherapy, and immunotherapy,^11^, ^12^ especially the role of immune checkpoint inhibitors.^13^ Oh et al.^14^ identified significant differences in gut microbial diversity and composition between immunotherapy‐responsive and immunotherapy‐nonresponsive patients. Emerging research underscores the vital influence of the gut microbiota, comprising microorganisms and their metabolites, on the immune system, thereby affecting the efficacy of immune checkpoint inhibitors (ICIs).^15^ This study, inspired by the lung‐intestinal axis and prior literature, investigates the alterations in intestinal microecology and immune function in advanced unresectable lung adenocarcinoma patients experiencing varied immunotherapy outcomes. It aims to unveil novel strategies for the effective management and prolonged survival of patients with advanced lung cancer.

## 2 INFORMATION AND METHODS

This study was carried out from January 1, 2021, to January 1, 2023, enrolling a total of 30 patients with lung adenocarcinoma, treated at the Oncology Center of Qingdao Hiser Hospital affiliated with Qingdao University. Eligible participants were those with stage IV inoperable nondriver gene lung adenocarcinoma, confirmed via definitive pathology, immunohistochemistry, and genetic testing. The inclusion criteria included: ① newly diagnosed patients meeting the diagnostic criteria who had not received any prior treatment; ② individuals aged between 18 and 65 years, regardless of gender; ③ patients with a PD‐L1 tumor proportional score (TPS) of ≥50%; ④ patients who consented to participate in the study and signed the informed consent form; ⑤ epidermal growth factor receptor (EGFR) wild type. Exclusion criteria encompassed individuals with serious respiratory, circulatory, digestive, urinary, endocrine, blood, neurological diseases, or psychiatric disorders. (“Serious” requires hospitalization or requires extended hospitalization, interferes with work, ability to live, inability to communicate on one's own, life‐threatening or death‐threatening, and is unable to follow planned antitumor therapy.) Additionally, pregnant and lactating individuals were excluded, as well as those who had taken antibiotics or micro‐ecological preparations (micro‐ecological preparations, also known as antibiotics, biotin, live bacterial preparations, and probiotics, are biological preparations containing live bacteria used in animals). These preparations are derived from animals or nature, identified or artificially created through bioengineering, and undergo specialized processes like cultivation, fermentation, drying, and processing. Animal microecological preparations encompass probiotics (bacteria) as well as substances like oligosaccharides that promote the growth and reproduction of normal microbial groups. Essentially, any preparation that fosters the growth of normal microorganisms while inhibiting pathogenic bacteria is termed a ‘microecological preparation’ within the last month or had undergone early surgical procedures (all patients with stage IIIB and prior lung cancer who are feasible for radical surgical resection or who are feasible for radical surgical resection after neoadjuvant therapy) or feasible radical radiotherapy. Patients positive for driver genes were also excluded. Discontinuation and dropout criteria included individuals who (1) took drugs prohibited by the study; (2) experienced serious adverse events (SAE); (3) did not complete the required cycles of observation; or (4) missed a visit during the follow‐up period. The study was approved by the Ethics Committee of Qingdao Hiser Hospital, and informed consent was obtained from all participants. Ethics Committee approval number: 2021HC01LS022.

Serious adverse event (SAE) is defined as an event during the course of the study that requires hospitalization, prolongs hospitalization, is disabling, affects the ability to work, is life‐threatening or fatal, or results in a congenital malformation.

### 2.1 Treatment protocol

Patients received the standard single‐agent sintilimab immunotherapy at a dosage of 200 mg every 21 days (q21d).

Observation indicators included T lymphocyte subpopulations and intestinal flora. Peripheral elbow vein blood (2 mL) was drawn from fasting patients between 7:00 AM and 9:00 AM before treatment and on the day of the completion of four treatment cycles, with sodium heparin (100 μL) added as an anticoagulant. Subsequently, 20 μL of the corresponding antibody was mixed with the anticoagulated blood sample, and the reaction was allowed to proceed for 20 min. After adding 500 μL of erythrocyte lysate and standing for 15 min at room temperature, 500 μL of phosphate buffer was added, thoroughly shaken, and allowed to stand for another 20 min. Levels of CD3+, CD4+, and CD8+ were detected using a Navios 6 COLORS/2 flow cytometer (Beckman Coulter Ireland, Inc.) following a 20‐min reaction. The composition of the intestinal flora was determined through 16S rRNA gene sequencing technology. Approximately 3 g of fecal samples was collected from patients both before treatment and 24 h after completing 4 cycles of treatment. To prevent sample contamination, subjects were instructed to empty their bladders before collecting feces. Researchers wearing disposable sterile gloves carefully collected fresh feces from the middle and inner parts of the sample to minimize external contamination. These samples were immediately placed into fecal genome protection solution (provided by Nanjing Ovison Gene Technology Co.) for pretreatment. The samples were then divided into three equal parts, sealed in sterile freezing tubes and stored in liquid nitrogen for uniform detection. High‐throughput sequencing and bioinformatics analysis were conducted by Nanjing Ovison Gene Technology Co.

### 2.2 Evaluation grouping criteria

The efficacy of the treatment was assessed after four cycles using the RECIST 1.1 criteria for solid tumor efficacy. The treatment response group was categorized into stable condition (SD), stable condition with insufficient tumor shrinkage for remission (SD−), partial remission (PR), and complete remission (CR). The nonresponse group was categorized into partial progression (PD) and stable condition with insufficient tumor progression for partial progression (SD+).

### 2.3 Statistical methods

The data were analyzed using SPSS 26.0. Descriptive statistics were used to describe the measurement data (mean ± standard deviation). The paired‐sample *t*‐test was employed for data conforming to a normal distribution, while the rank‐sum test was used for data not conforming to a normal distribution. A *p*‐value of less than 0.05 was considered statistically significant.

## 3 RESULTS

### 3.1 General information

This study enrolled 30 patients diagnosed with advanced lung adenocarcinoma, evenly divided into a control group and a treatment group, comprising 18 males and 12 females. The participants had an average age of 56.15 ± 6.32 years and a mean Body Mass Index (BMI) of 22.03 ± 2.62 kg/m^2^. Notably, one case of attrition was observed after completing four cycles of the trial.

### 3.2 Immune function analysis

In the group that responded to treatment, a significant increase in CD4+ T cell counts was observed after four treatment cycles, alongside a reduction in CD8+ T cell levels (*P* < 0.05), indicating a positive shift in immune function. Conversely, in the nonresponsive group, a significant decrease in the proportion of CD8+ T cells was noted both before and after treatment, suggesting the influence of anti‐tumor immunotherapy on immune parameters. Detailed findings are presented in Tables 1 and 2.

**TABLE 1:** Comparison of T‐lymphocyte subpopulation expression rates before and after treatment in 15 patients in the treatment‐responsive group (x̅ ± s).

Detection indicators	Treatment‐responsive group pretreatment	Treatment‐responsive group posttreatment	T	*P*
CD4^+^T (cells/mL)	564.40 ± 330.28	770.87 ± 313.66	−2.67	0.027
CD8^+^T (cells/mL)	712.27 ± 292.63	490.07 ± 157.67	4.40	<0.001
CD4/CD8	0.85 ± 0.47	1.59 ± 0.45	−5.67	0.13

**TABLE 2:** Comparison of cell expression rates of T lymphocyte subpopulations before and after treatment in 15 patients in the treatment‐nonresponsive group (x̅ ± s).

Detection indicators	Treatment‐nonresponsive group pretreatment	Treatment‐nonresponsive group posttreatment	T	*P*
CD4^+^T (cells/mL)	598.67 ± 299.54	606.67 ± 299.18	−0.67	0.514
CD8^+^T (cells/mL)	659.27 ± 322.59	534.93 ± 269.47	2.66	0.019
CD4/CD8	1.03 ± 0.42	1.29 ± 0.62	−2.17	0.047

### 3.3 Intestinal flora comparison

For specimen analysis, we labeled the pretreatment samples from the nonresponsive group as MYA1 and MYA2 and the pretreatment and posttreatment samples from the responsive group as MYB1 and MYB2, respectively.

#### 3.3.1 OTUs analysis

**FIGURE 1:**
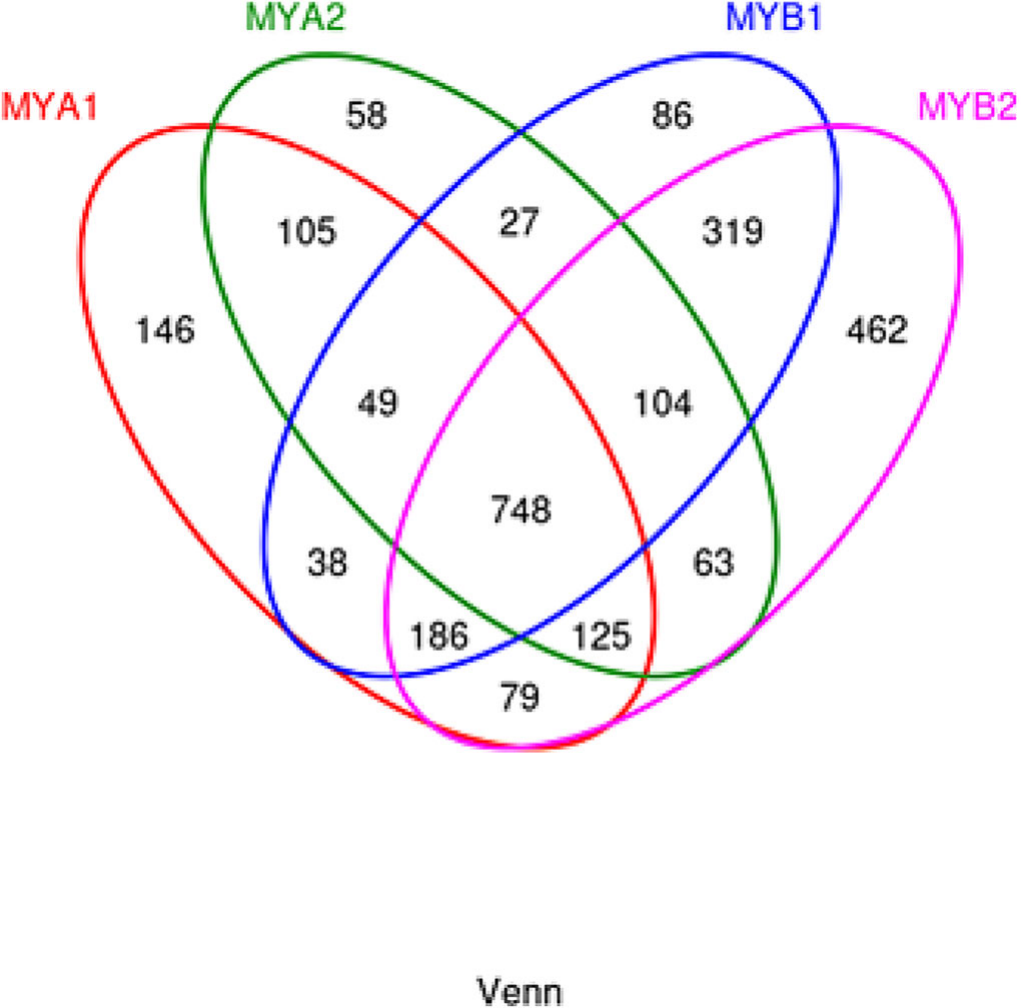
Venn diagram illustrating the distribution of OTUs. Each color represents a different sample. The overlapping area of the circles corresponds to the intersection of OTUs shared among multiple samples, while the nonoverlapping parts represent unique OTUs specific to each sample.

The investigation produced a total of 2705 operational taxonomic units (OTUs), with 2595 remaining after normalization. Within these, 146 OTUs were identified in MYA1, 58 in MYA2, 86 in MYB1, and an elevated number of 462 in MYB2, totaling 748 OTUs across all groups (see Figure 1 for reference). OTUs serve as unique identifiers assigned to specific taxonomic categories (such as lineage, genus, species, subgroup, etc.) in phylogenetic or population genetic analyses, aiding in the streamlined assessment of microbial diversity.

#### 3.3.2 Alpha diversity analysis

**FIGURE 2:**
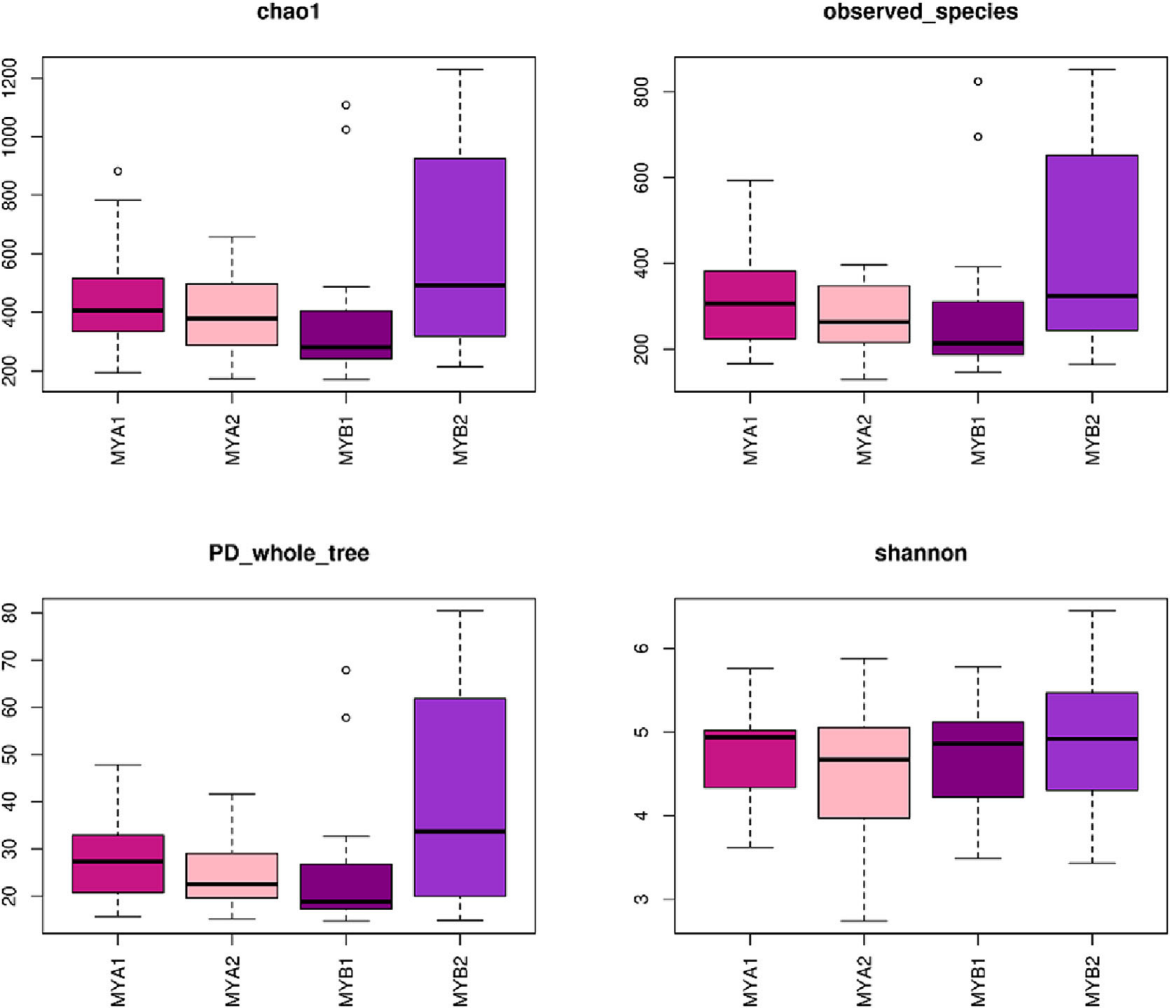
Box plot of alpha diversity index.

The Chao1 index, a measure of strain richness, estimates the total number of operational taxonomic units (OTUs) within a community (Figure 2). The Simpson index, accounting for both diversity and uniformity, serves as a microbial diversity indicator in samples. PD_whole_tree, a phylogenetic diversity index, considers species abundance and evolutionary distance, with higher values indicating greater community diversity. The Shannon index, another measure for estimating microbial diversity within samples, suggests higher community diversity with increased values. Observed_species denotes the count of OTUs observed as sequencing depth intensifies. Goods_coverage represents the observation depth. Our analysis identified statistically significant differences in three diversity indices (Chao1, Observed_species, and PD_whole_tree) between pretreatment and posttreatment samples in the treatment‐responsive group (see Figure 2). These results indicate changes in the diversity of human intestinal flora in patients from the treatment‐responsive group following treatment.

#### 3.3.3 Beta diversity

**FIGURE 3:**
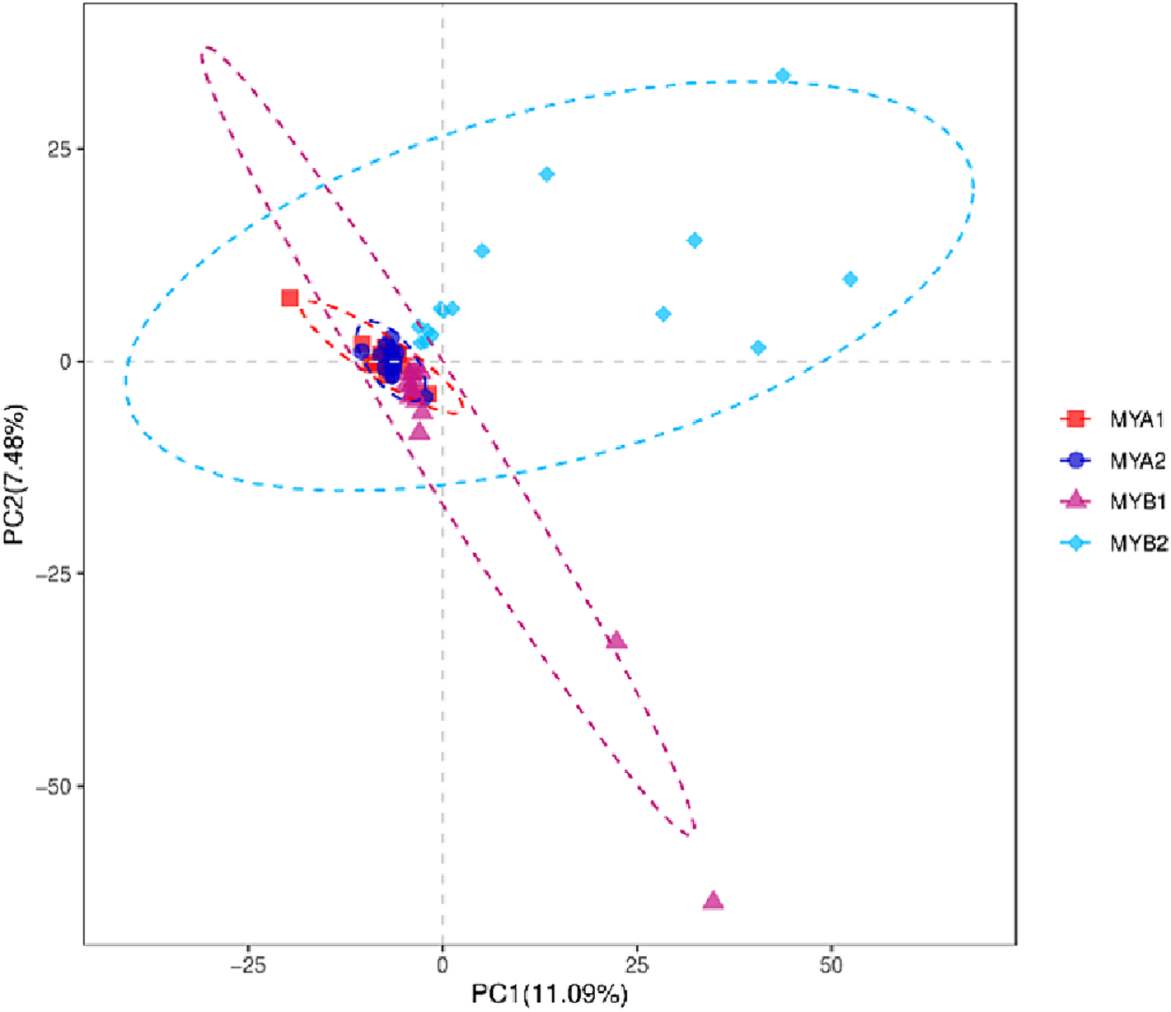
PLS‐DA analysis shows that there is no overlap between the samples from the treatment response group and the other groups. This indicates a significant change in the microbial community of the samples after treatment.

Partial least squares discrimination analysis (PLS‐DA) is a multivariate statistical method used for discriminant analysis. Discriminant analysis is a common statistical technique used to categorize research objects based on observed or measured variables. PLS‐DA is a supervised method that models the relationship between microbial content and sample categories to predict the category of a given sample (Figure 3).

#### 3.3.4 Species composition analysis

**FIGURE 4:**
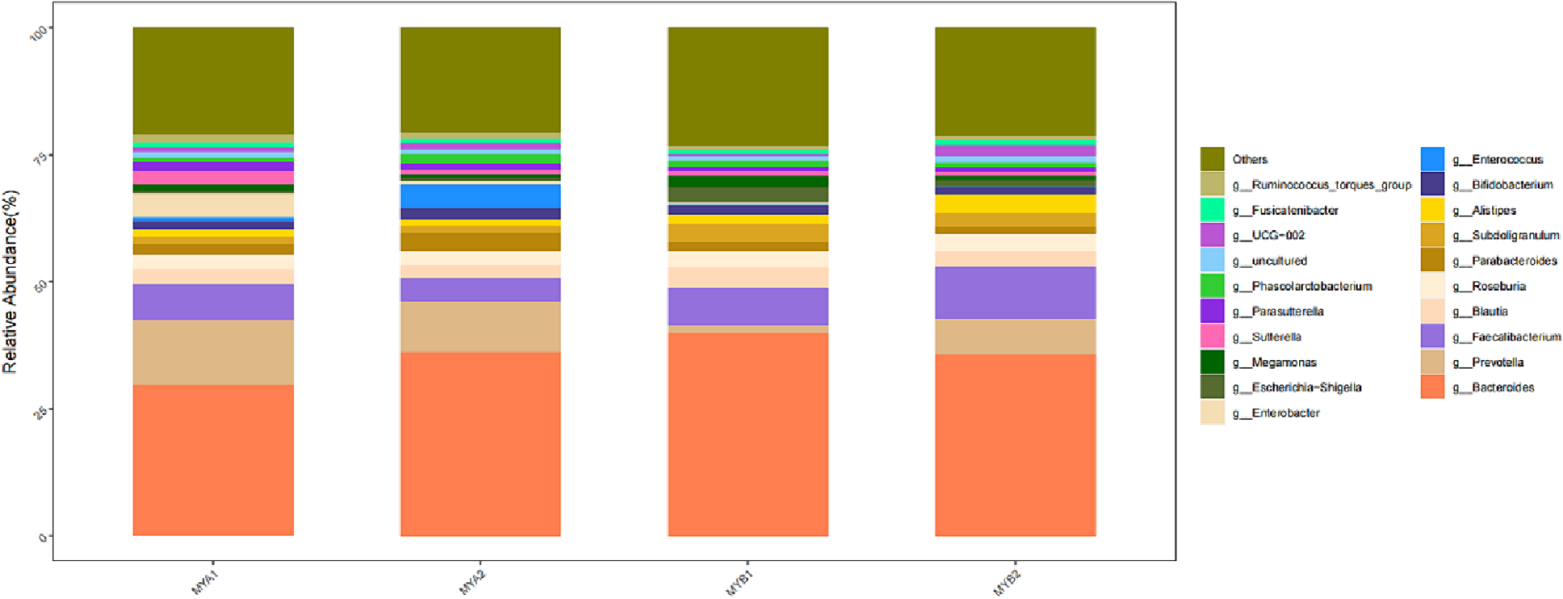
Histogram of dominant flora at the phylum classification level.

**FIGURE 5:**
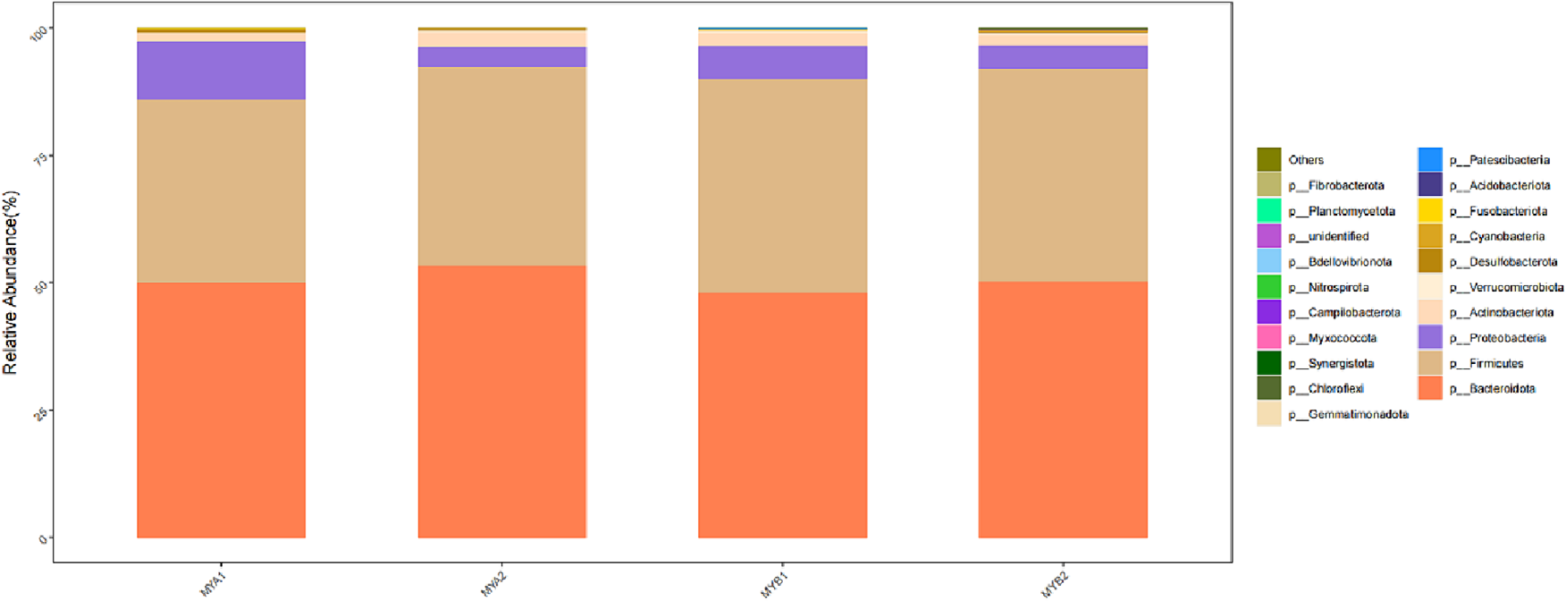
Histogram of dominant flora at genus taxonomic level.

**FIGURE 6:**
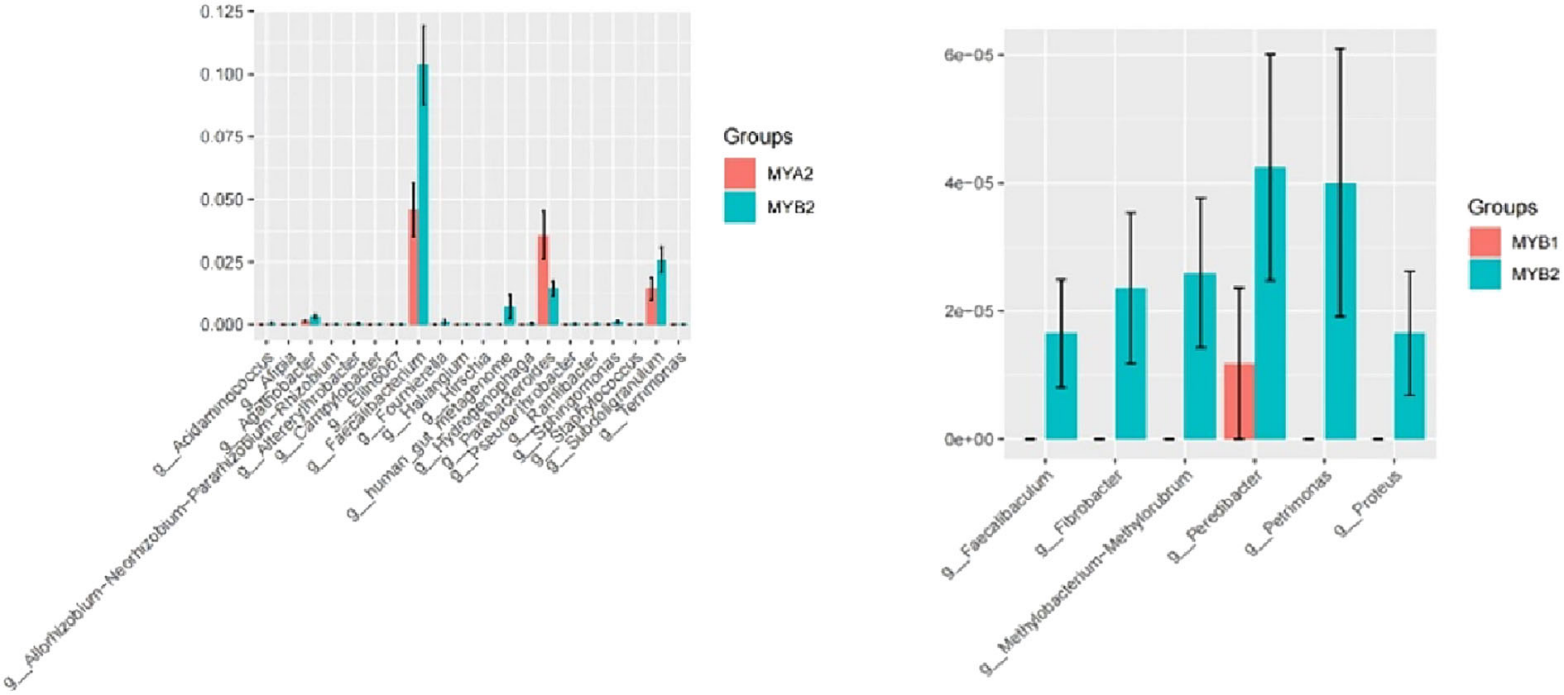
Histogram of species differences in different groups.

In our study, 2595 OTUs underwent species annotation, revealing insights into the microbial landscape at the phylum level (illustrated in Figure 4). The combined prevalence of Bacteroidetes, Firmicutes, Proteobacteria, and Actinobacteria constituted 90% of the gut microbiome across the cohort, marking them as the primary phyla. Comparative assessment between the pretreatment and posttreatment samples (MYA1/MYB1 vs. MYA2/MYB2) highlighted a notable increase in Proteobacteria from 50.01% and 48.13% to 53.30% and 50.16%, respectively. Conversely, Actinobacteria abundance diminished from 11.22% and 6.39% to 3.79% and 4.54%, respectively, following treatment. Specifically, the MYB2 samples demonstrated a decreased proportion of thick‐walled to anamorphic phyla relative to MYB1. Delving into genus‐level distinctions (as seen in Figure 5), genera such as Bacteroides, Prevotella, Faecalibacterium, Blautia, and Roseburia stood out due to their elevated representation, denoting them as dominant genera. A granular analysis at the genus level underscored a significant depletion in g__Butyricoccus for MYA2 compared to MYA1. Conversely, MYB2 showed a remarkable enhancement in several genera compared to MYB1, despite the overall lower abundance. Importantly, MYB2 exhibited a pronounced increase in the beneficial genera g__Faecalibacterium and g__Subdoligranulum against MYA2. Moreover, these beneficial bacteria were significantly more abundant in MYB2 compared to MYA2. A significant downturn was recorded in the genus g__Parabacteroides. Detailed observations are available in Figure 6.

## 4 DISCUSSION

This study delves into the pivotal role of the human gut microbiota and its correlation with lung cancer. The human gut microbiota comprises bacteria, archaea, viruses, and eukaryotic microbes, boasting approximately 150 times the number of genes found in the human genome, hence often referred to as the “second genome of the human body.”^16^ It plays a crucial role in maintaining human health by interacting with various organs such as the kidneys, brain, cardiovascular system, and skeletal system. Notably, the intricate connection between the lungs and the gut has spurred significant research into the relationship between the gut microbiota and lung diseases in recent years. Prior studies have underscored a close association between the composition and dysbiosis of the gut microbiota and various lung diseases, including asthma, chronic obstructive pulmonary disease (COPD), idiopathic pulmonary fibrosis, lung infections, and lung cancer.^17^, ^18^ These comorbidities may significantly influence treatment outcomes and patient survival rates. For instance, comorbidities like cardiovascular diseases, diabetes, and chronic kidney diseases may elevate surgical risks, affect immunotherapy tolerance, or impact patient survival rates through diverse pathways. Therefore, it is imperative to consider the presence and impact of comorbidities in interpreting research findings.

Based on the gut‐lung axis theory, this study aims to explore the correlation between the gut microbiota and lung cancer. However, further research is warranted to explore effective methods for modulating lung cancer development, reducing toxicity, and enhancing the efficacy of conventional treatments. Ultimately, these endeavors strive to improve patient survival rates and enhance their quality of life.

Our study scrutinized alterations in the gut microbiota and immune function in patients with advanced unresectable lung adenocarcinoma exhibiting varied immunotherapy outcomes. Beta diversity analysis revealed no significant differences between samples in the MYA1 and MYA2 groups, while notable distinctions were observed between samples in the MYB1 and MYB2 groups, indicating changes in gut microbiota composition in the treatment response groups. Concerning species composition, at the phylum level, we noted abundance comparisons between MYA1, MYB1, MYA2, and MYB2 groups. Results unveiled an increase in Firmicutes phylum abundance and a decrease in Bacteroidetes phylum abundance posttreatment, suggesting treatment‐induced adjustments in microbial community structure and enhanced abundance of beneficial phyla. Notably, the MYB2 group exhibited a reduced Actinobacteria/Firmicutes ratio compared to the MYB1 group, though the underlying mechanism remains unclear. At the genus level, Prevotella, Faecalibacterium, Bacteroides, Blautia, and Roseburia emerged as major genera, yet their dominant mechanisms remain elusive. Differential species analysis unveiled a significant decrease in Butyricoccus levels in the MYA2 group relative to the MYA1 group. Conversely, multiple genera in the MYB2 group exhibited higher levels compared to the MYB1 group, albeit relatively lower. Additionally, the MYB2 group evidenced a notable increase in Faecalibacterium and Bifidobacterium genera compared to the MYA2 group, both being beneficial bacteria. Conversely, the MYB2 group registered a marked decrease in Actinobacteria genus. Enhancing gut microbiota structure holds promise for augmenting treatment outcomes. Nonetheless, it is crucial to acknowledge that this study's preliminary gut microbiota screening analysis relied on a small sample size, necessitating further studies with larger sample sizes to validate these findings.

Numerous studies^19^, ^20^, ^21^ have underscored the close relationship between tumors and the immune system, with the immune microenvironment significantly impacting tumor occurrence, progression, and distant metastasis. Among immune cells, T lymphocytes‐mediated cellular immunity, particularly CD4+ and CD8+ T lymphocytes, play dominant roles. CD4+ T cells primarily function as helper T cells, supporting anti‐tumor immunity, while CD8+ T cells serve as cytotoxic T lymphocytes, inducing tumor cell apoptosis through diverse pathways. The CD4+/CD8+ ratio crucially determines tumor patients' cellular immune function, with an increased ratio indicating dominant positive immune response regulation, while a decreased or less than 1 ratio suggests impaired cellular immune function. Past research^18^, ^22^ has validated the pivotal role of the gut microbiota in lung cancer occurrence, immunity, and immunotherapy. Moreover, lung microbiota's influence on these factors has been substantial. It is noteworthy that the gut microbiota is not solely influenced by medications but can also affect individual drug responses by altering drug structure via drug enzymes, consequently impacting drug bioavailability, bioactivity, and toxicity. Thus, these alterations indirectly affect individual responses to tumor immunotherapy. Our study's results indicated significantly higher levels of CD4+ T cells and CD4+/CD8+ ratios in the treatment group compared to baseline levels before treatment. Additionally, the treatment group exhibited lower CD8+ T cell levels. This suggests that immunotherapy can effectively modulate peripheral blood T lymphocyte subset distribution. Immunotherapy may induce immune regulation, affecting immune cell infiltration and activity within the tumor microenvironment. In this scenario, treatment may lead to decreased CD8+ T cells within tumor tissues, partly elucidating the decrease in CD8+ T cell proportion in the nonresponsive group. Moreover, immunotherapy may affect immune cell numbers and functions through alternative mechanisms, such as regulating T cell activation and proliferation, as well as modulating immune suppressor cell functions. Therefore, the alterations in CD8 + T cell proportions pretreatment and posttreatment may be treatment‐induced, reflecting immunotherapy's impacts on the immune system.

Gut microbiota metabolic pathways exert significant influence on eliciting immune therapy responses. Furthermore, the gut‐lung axis plays a crucial role in shaping immune responses and may impact respiratory disease progression. Spacova et al.'s^23^ study observed a substantial reduction in airway inflammation in an asthma mouse model upon introducing lactobacilli strains, compared to mice without such strains. This finding underscores the gut‐lung axis'^24^ potential in alleviating inflammatory responses during pneumonia. Dysregulation of the gut microbiota may result in gut‐lung axis‐induced lung inflammation, thereby promoting abnormal cell proliferation and ultimately affecting immunotherapy efficacy.

Recent research^25^ unveiled that non‐small cell lung cancer (NSCLC) patients responding to nabumab (a PD‐1 inhibitor) harbored a more diverse baseline gut microbiota composition. Moreover, their microbiota composition remained stable throughout treatment. Patients with high microbiota diversity experienced significantly prolonged progression‐free survival (PFS) compared to those with low diversity. Subsequent studies^26^ identified an enrichment of mucinophilic Ackermannia in the gut microbiota of NSCLC or renal cell carcinoma patients responding to immunotherapy upon initial diagnosis. Additionally, two studies explored the relationship between baseline gut microbiota composition/diversity and immune checkpoint inhibitors' (ICIs) efficacy in NSCLC patients, reporting correlations between gut microbiota composition/diversity and ICI efficacy. Identifying gut microbiota biomarkers associated with early progression and long‐term benefit in patients can aid in personalized cancer patient management.

Clinical data advocate modulating the microbiota as a promising avenue to enhance cancer immunotherapy effectiveness, especially for CTLA‐4 and PD‐1 immune checkpoint inhibitors. Fecal microbiota transplantation (FMT), entailing introducing feces from healthy individuals into patients' gastrointestinal tracts, aims to restore or rebalance gut microbiota composition. Previous studies^27^ demonstrated that germ‐free mice receiving fecal transplants from immunotherapy responders exhibited heightened sensitivity to PD‐1 inhibitors compared to those receiving transplants from nonresponders. Future understanding of the gut microbiota‐tumor relationship will continue to evolve. The gut‐lung axis theory underscores the importance of viewing the human body holistically and recognizing the significance of gut‐lung homoeostasis in clinical practice. The gut microbiota's intertwined relationship with immune regulation and cancer development is crucial in controlling tumor immune evasion and immune sensitivity. Its impact on tumor immunotherapy has been corroborated in animal experiments and clinical studies. Additionally, leveraging multi‐omics technologies to study gut microbiota dysbiosis' genetic and molecular mechanisms and its effects on cancer development and treatment is essential. By exploring from micro and macro perspectives, we can actively seek strategies to modulate gut microbiota balance. Standardizing gut microbiota dysbiosis experimental animal model modeling and testing standards is imperative to minimize deviations from the human body and avoid potential issues. Furthermore, additional experiments should ascertain gut microbiota's positive rates as clinical disease diagnostic biomarkers. However, several aspects^28^, ^29^ remain undiscovered in this field. For instance, optimizing treatment outcomes by avoiding unnecessary antimicrobial drug use and maintaining microbial community homeostasis is crucial in anti‐tumor therapy. Future investigations into the potential mechanisms of these complex interactions and identification of specific microbes crucial in mediating anti‐tumor responses and overall cancer development are essential. Understanding gut microbiota and its metabolites' biological mechanisms in response to cancer treatment and immunotherapy is pivotal for enhancing ICI (immune checkpoint inhibitor) therapy efficacy.^30^, ^31^ This comprehension will enable rational modulation of microbial activity. Such experiments can help unearth more connections between the gut microbiota and lung cancer. Additionally, integrating the “three‐factor” principle based on individuals, time, and location with the backdrop of precision medicine may enhance outcomes for more lung cancer patients.

Nevertheless, this study harbors several limitations. For instance, a small sample size issue may impede result generalizability. Additionally, limitations in study design or methodology may introduce biases or overlook factors.

## AUTHOR CONTRIBUTIONS

Jin Tian and Shuang He are in charge of data acquisition and data analysis, Jianhua Zang, Lin Long, and Peng Liu are in charge of literature review, clinical data collection, and validation, and Shuang He made a contribution to manuscript editing. Yexi Zhang and Jun Xiao are in charge of manuscript review. All authors read and approved the final manuscript.

## CONFLICT OF INTEREST STATEMENT

The authors declare no conflict of interest.

## ETHICS STATEMENT

The study was approved by the Ethics Committee of Qingdao Hiser Hospital, informed consent was obtained from all subjects. Informed consent was obtained from all individual participants included in the study.

## Data Availability

The datasets generated during and/or analyzed during the current study are available from the corresponding author on reasonable request.
